# Activities Under the RAISE Family Caregiving Act: Developing a National Strategy to Support Family Caregivers

**DOI:** 10.1093/geroni/igab046.244

**Published:** 2021-12-17

**Authors:** Wendy Fox-Grage

**Affiliations:** NASHP, Washington, DC, District of Columbia, United States

## Abstract

This presentation describes the unique collaboration between The John A. Hartford Foundation, the Administration or Community Living (ACL), and the National Academy for State Health Policy (NASHP) in supporting the RAISE Act Family Caregiver Resource and Dissemination Center, and the goals and activities of the RAISE Act Family Caregiving Advisory Council. Most importantly, she will present the the development of recommendations for a national strategy to support family caregivers involving all levels of government as well as private-sector actors. These recommendations fall into five primary areas, which Fox-Grage will discuss in detail. She will also discuss the Center’s development of family caregiving resources for state and federal policymakers and other stakeholders as well as next steps in turning the Council’s recommendations into concrete action.

